# Cultivar-Dependent Thermal Flesh Breakdown in Apple Associated with Cell Wall Polysaccharide Modification, with Pronounced Effects in Cooking Apple ‘Bramley’s Seedling’

**DOI:** 10.3390/foods15081375

**Published:** 2026-04-15

**Authors:** Mitsuho Nakagomi, Tomomichi Fujita, Saki Sato, Akari Oka, Jong-Pil Chun, Kazuhiro Matsumoto

**Affiliations:** 1 The United Graduate School of Agricultural Science, Gifu University, 1-1 Yanagido, Gifu 501-1193, Japan; birodo.hikari.2@gmail.com (M.N.); oka12125@gmail.com (A.O.); 2 Faculty of Agriculture, Shizuoka University, 836 Ohya, Suruga, Shizuoka 422-8531, Japan; 3 Fujisaki Farm, Faculty of Agriculture and Life Science, Hirosaki University, 7-1 Shimotabi, Fujisaki, Aomori 038-3802, Japan; fujita@hirosaki-u.ac.jp (T.F.); sa110006@hirosaki-u.ac.jp (S.S.); 4 Department of Horticulture, Chungnam National University, Daejeon 305-764, Republic of Korea; jpchun@cnu.ac.kr

**Keywords:** fruit softening, thermal processing, texture, pectin solubilization, pectin degradation, ‘Fuji’, varietal diversity

## Abstract

Heat-induced softening of apple fruit varies markedly among cultivars; however, the biochemical factors underlying these differences remain incompletely understood. This study investigated the relationship between cell wall modifications and thermal flesh breakdown in three apple cultivars (‘Bramley’s Seedling’, ‘Fuji’, and ‘Toki’). Fruit flesh samples were heated under controlled conditions and analyzed for changes in texture properties, cell structure, cell wall composition, and molar mass distribution. Heating increased water-soluble pectin in all cultivars, with a markedly greater increase in ‘Bramley’s Seedling’, indicating pronounced pectin solubilization during thermal treatment. A pronounced shift from high- to low-molar-weight polymers in the Na_2_CO_3_-soluble fraction was also observed only in ‘Bramley’s Seedling’, suggesting extensive depolymerization of the Na_2_CO_3_-soluble pectic polymers. A decrease in hemicellulose and cellulose content following heating was observed exclusively in ‘Bramley’s Seedling’. Consistently, this cultivar exhibited significantly lower gumminess and chewiness compared with the other cultivars. Beyond compositional changes, ‘Bramley’s Seedling’ exhibited severe tissue disintegration and distinctive rheological behavior indicative of extensive cell rupture. In contrast, ‘Fuji’ and ‘Toki’ retained relatively stable cell wall structures and maintained tissue integrity after heating. These findings suggest that cultivar-dependent disassembly of cell wall polysaccharides, particularly pectin depolymerization and solubilization, is strongly associated with heat-induced tissue breakdown.

## 1. Introduction

Apples (*Malus domestica* Borkh.) are one of the most widely cultivated fruit crops worldwide, consumed both fresh and in a variety of processed products, including juices, purees, jams, and baked goods [1]. Beyond differences in flavor and acidity, apple cultivars exhibit substantial variation in texture and processing behavior [2,3]. In particular, some cultivars, such as ‘Bramley’s Seedling’, rapidly lose their structural integrity during heating, whereas others, such as ‘Calville Blanc d’Hiver’, retain their structural form. Such cultivar-dependent differences strongly influence the suitability of apples for specific culinary and industrial processing applications.

The mechanical properties of apple flesh are largely determined by the structure of the plant cell wall, which primarily consists of cellulose, hemicellulose, and pectin [4,5]. Cellulose microfibrils provide the load-bearing framework of the wall, hemicelluloses connect these microfibrils and contribute to wall rigidity, and pectins—especially homogalacturonan located in the middle lamella—play a key role in cell-to-cell adhesion through calcium-mediated cross-linking [4,6]. Consequently, modifications of these polysaccharides are closely associated with fruit softening and tissue disintegration.

During fruit ripening, softening is commonly associated with pectin solubilization and depolymerization. These changes are typically characterized by an increase in water-soluble pectin and a decrease in more tightly bound pectin fractions, such as EDTA- and Na_2_CO_3_-soluble pectin. Such modifications have been widely reported in various climacteric and non-climacteric fruits, including peach [7], kiwifruit [8], apple [9], and strawberry [10].

Similar modifications of cell wall polysaccharides have also been observed during thermal processing. Heating promotes pectin solubilization and depolymerization, as well as weakening of the cellulose–hemicellulose network, leading to a loss of tissue firmness and structural cohesion in fruits and vegetables [11]. For example, thermal processing of apples has been reported to increase water-soluble pectin and reduce the molar mass of pectic polymers, accompanied by changes in tissue microstructure and rheological properties [12,13,14]. In addition to pectin modification, changes in hemicellulose and cellulose may also contribute to tissue disintegration during heating, although their roles in cultivar-dependent thermal breakdown remain poorly understood.

**Figure 1:**
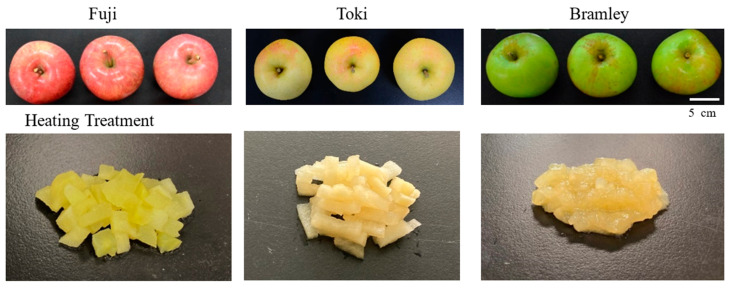
Comparison of external appearance and heated flesh among three apple cultivars: ‘Fuji’, ‘Toki’, and ‘Bramley’s Seedling’ (hereafter referred to as Bramley).

‘Bramley’s Seedling’ is a traditional cooking cultivar originating in the United Kingdom, widely recognized for its rapid collapse into a puree-like texture during heating, a behavior that markedly contrasts with that of dessert cultivars (Figure 1). Such rapid softening is uncommon among apple cultivars, making ‘Bramley’s Seedling’ a valuable model for investigating extreme thermal breakdown behavior. However, the structural and biochemical mechanisms underlying this distinct thermal response have not been fully elucidated, particularly with respect to coordinated changes across pectin, hemicellulose, and cellulose fractions.

To address these issues, the present study focuses on cultivar-dependent mechanisms of thermal breakdown through a comparative approach using ‘Bramley’s Seedling’ and two dessert cultivars, ‘Fuji’ and ‘Toki’ (Figure 1). ‘Fuji’ is one of the most widely cultivated dessert apples worldwide, characterized by its crisp texture and long storability. ‘Toki’, which also exhibits a crisp texture suitable for fresh consumption, was additionally selected because its commercial harvest period more closely approximates that of ‘Bramley’s Seedling’ than does that of ‘Fuji’.

By integrating compositional, molar weight, and structural analyses of cell wall polysaccharides in these cultivars, this study aims to elucidate cultivar-specific mechanisms of cell wall disassembly during thermal processing, with particular emphasis on the exceptional behavior of ‘Bramley’s Seedling’.

## 2. Materials and Methods

### 2.1. Plant Materials

Fruits of three apple cultivars—‘Fuji’, ‘Toki’, and ‘Bramley’s Seedling’—were used in this study. ‘Fuji’ and ‘Toki’ were each harvested from a single tree, whereas ‘Bramley’s Seedling’ was harvested from two trees, owing to differences in orchard availability. ‘Fuji’ and ‘Toki’ were cultivated at the Fujisaki Farm of Hirosaki University, located in Fujisaki, Aomori, Japan (40°39′25″ N, 140°29′9″ E). Both trees were 24 years old in 2020, grafted onto *Malus prunifolia* rootstocks, and trained to a central leader form (5.0 × 3.5 m spacing). ‘Bramley’s Seedling’ was cultivated at the contracted orchard of Raguno Sasaki Co. (Aomori, Japan), located in Hirosaki, Aomori, Japan (40°33′34″ N, 140°28′44″ E). ‘Bramley’s Seedling’ trees were 15 years old in 2020, grafted onto *Malus prunifolia* rootstocks, and trained to a central leader form (5.0 × 6.0 m spacing).

Fruits of all three cultivars were sampled in the 2020 and 2021 seasons to account for potential year-to-year variation. ‘Fuji’ was harvested on 6 November 2020 (180 days after full bloom; DAFB) and 5 November 2021 (182 DAFB). ‘Toki’ was harvested on 2 October 2020 (145 DAFB), and 1 October 2021 (147 DAFB). ‘Bramley’s Seedling’ was harvested on 11 September 2020 (125 DAFB) and 10 September 2021 (127 DAFB). The harvest date for each year corresponded to the commercial harvest time for each cultivar. Fruits were randomly sampled prior to analysis. For each cultivar, six fruits were used as biological replicates (*n* = 6). Fruits with severe physical injuries were excluded from the analysis. Details of sample allocation and replication are described in each section below.

### 2.2. Fruit Quality Measurement

All harvested fruits were immediately transported to the Faculty of Agriculture, Shizuoka University, at 4 °C. Quality measurements were performed on six individual fruits per cultivar (biological replicates, *n* = 6). Fruit fresh weight was measured using a digital balance. Fruit length and diameter were measured using digital calipers, and the length-to-diameter (L/D) ratio was calculated. Skin color was measured using a color-difference meter (NF333; Nippon Denshoku, Tokyo, Japan). The starch content was determined by iodine staining with a potassium iodide–iodine solution and scored on a 1–5 scale (1 = low starch, 5 = high starch) according to the guidelines for apple production (The Public Foundation of the Aomori Apple Association, 2014). To measure ethylene production, each fruit was placed in an individual 1.45 L polypropylene jar, which was then sealed and held at 25 °C for 3 h to allow ethylene accumulation. After incubation, a 30 mL headspace gas sample was withdrawn from each jar using a handheld ethylene analyzer (F-950 Three Gas Analyzer; Felix Instruments, Camas, WA, USA). The headspace volume of each jar was corrected by subtracting the fruit volume, which was determined by water displacement. Ethylene production was expressed as µL kg^−1^ h^−1^. The soluble solids content (SSC) of the juice was measured using a digital refractometer (N-1; Atago, Tokyo, Japan), and the total titratable acidity was determined by titration with 0.1 N NaOH and expressed as malic acid equivalents.

### 2.3. Assessment of Flesh Firmness and Texture Profile Analysis

Flesh firmness and texture profile analysis were each conducted using six individual fruits as biological replicates (*n* = 6). The flesh firmness of unheated fruit was measured at two points on the equatorial region of each fruit after skin removal, using a hand-held penetrometer with an 11.1 mm diameter plunger (FT327; Facchini srl, Alfonsine, Italy).

For heated samples, each fruit was peeled and cut into 2 × 2 × 2 cm cubes. Each fruit cube was placed at the center of a 100 mL glass beaker, which was then positioned at the center of the microwave cavity. Samples were heated individually at 500 W for 30 s; these conditions were established through preliminary experiments in which heating time was systematically varied and sample temperature was monitored to achieve consistent softening. Therefore, this method was optimal for evaluating the characteristic textural softening, especially for ‘Bramley’s Seedling’. Firmness was measured using a texture analyzer (FRTS-50N; IMADA Co., Ltd., Toyohashi, Japan) equipped with a 5.0 mm diameter cylindrical plunger. One cube per fruit was measured (*n* = 6).

Texture profile analysis was performed on the heated flesh using the same texture analyzer. Cubic samples (2 × 2 × 2 cm) prepared following the same heating protocol were subjected to a double compression test using a 20.0 mm diameter cylindrical probe. The samples were compressed to a deformation distance of 7.0 mm at a crosshead speed of 2.0 mm s^−1^. Textural parameters, including viscosity, cohesiveness, adhesiveness, resilience, gumminess, and chewiness, were calculated from the resulting force–time curves using the manufacturer’s software (Force Recorder Professional, version 1.01; IMADA Co., Ltd., Toyohashi, Japan). However, as the hardness of the ‘Fuji’ decreased only slightly upon heating and did not fall below the measurement limit (50 N), it could not be measured using this device.

### 2.4. Observation of the Fruit Tissue Structure

Tissue structure was examined in six individual fruits (*n* = 6). The cell structures of both unheated and heated apple flesh were observed using a scanning electron microscope (TM-3030Plus; Hitachi, Tokyo, Japan) in low-vacuum mode at 5 kV. Small tissue blocks (approximately 1 × 2 × 5 mm) were excised radially from beneath the epidermis, and the freshly cut surfaces were examined without conductive coating, consistent with the low-vacuum imaging mode. Heated samples were prepared in the same manner as described in Section 2.3 (microwave heating at 500 W for 30 s).

### 2.5. Cell Wall-Bound Ca Content Measurement

Cell wall-bound Ca content was measured using three individual fruits as biological replicates (*n* = 3). Flesh samples were stored at −60 °C until analysis. Samples were freeze-dried for 72 h, and 0.5 g of the powdered tissue was ashed in a muffle furnace at 500 °C for 6 h. The resulting ash was dissolved in 0.01 N HCl according to the method of Choi [15]. Ca concentration was determined using an atomic absorption spectrophotometer (AA-7000; Shimadzu, Kyoto, Japan).

### 2.6. Preparation of Ethanol-Insoluble Solids (EIS) and Extraction of Cell Wall Components

EIS were prepared from three individual fruits (biological replicates, *n* = 3), with extractions performed in triplicate per fruit (technical replicates), yielding nine measurements in total. Apple flesh samples were peeled and cut into small pieces. Aliquots of 10 g were placed into 50 mL polypropylene tubes and stored at −60 °C until analysis. To obtain the heated samples, we used the autoclave (MLS-2420; Sanyo Electric Co., Ltd., Tokyo, Japan) to ensure uniform heating without any loss of moisture or sample material. Based on preliminary experiments, apple flesh was sealed in individual polypropylene tubes and autoclaved at 105 °C for 3 min. The heating time referred to the holding time after the chamber reached the target temperature. All samples were processed under identical loading conditions in which heating time and temperature were systematically varied to achieve reproducible results under standardized conditions.

EIS was prepared using the method of Huber [10], with the following modifications. Ten g of tissue was added to 40 mL of 100% ethanol and homogenized at full speed for 3 min using a homogenizer (AHG-160A; AS ONE Corp., Osaka, Japan). The homogenate was boiled at 80 °C for 30 min to inactivate enzymes, washed with 80% ethanol, and filtered through glass microfiber filter paper (GF/C; Whatman, GE Healthcare, Tokyo, Japan). The residue was transferred to 100 mL of chloroform:methanol (1:1, *v*/*v*) and shaken for 30 min. After filtration, the residue was washed sequentially with 80% cold ethanol (three times) and 100% cold acetone (three times) and then dried at 38 °C for 24 h.

For cell wall component extraction, EIS samples from the three fruits were pooled and homogenized. All extractions were performed in triplicate (*n* = 3). Soluble pectin and hemicellulose fractions were prepared as described by Cheng and Huber [16] and Maclachlan and Brady [17], respectively, with some modifications. The EIS (100 mg) was sequentially extracted with 30 mL of each solvent: first with 90% dimethyl sulfoxide (DMSO), then with distilled water containing 0.2% sodium azide, subsequently with 50 mM EDTA (ethylenediaminetetraacetic acid) in 50 mM sodium acetate buffer (pH 6.5) for 12 h at 25 °C, and finally with 50 mM Na_2_CO_3_ containing 20 mM sodium borohydride for 12 h at 4 °C. Sodium borohydride was included in the Na_2_CO_3_ extraction to prevent β-elimination of pectic polymers during alkaline treatment. Each extraction step was carried out with continuous stirring. At the end of each extraction, the mixture was centrifuged (10,000× *g* for 10 min), and the supernatant was filtered through glass microfiber filter paper and Miracloth (Cat#475855; CALBIOCHEM, San Diego, CA, USA). This centrifugation and filtration procedure was repeated twice. The residues were subsequently extracted with 4% KOH and then 24% KOH, both containing 20 mM sodium borohydride, with shaking for 24 h at 25 °C. After centrifugation, the samples were filtered through glass microfiber filter paper and Miracloth. The Na_2_CO_3_, 4% KOH, and 24% KOH extracts were neutralized with acetic acid after filtration and adjusted to a final volume of 50 mL. The remaining residue was washed sequentially with 80% ethanol and 100% acetone (three times each) and dried at 38 °C for 24 h.

The uronic acid and total sugar contents in the pectin and hemicellulose fractions were determined using the m-hydroxydiphenyl method [18] and the phenol–sulfuric acid method [19], respectively.

### 2.7. Gel Permeation Chromatography of Cell Wall Pectic and Hemicellulose Fractions

Gel permeation chromatography was performed in triplicate (*n* = 3) for each extracted fraction. Water-, EDTA-, and Na_2_CO_3_-soluble pectins were applied to a Sepharose CL-6B-100 column (length 30 cm × internal diameter 1.4 cm; Sigma-Aldrich, St. Louis, MO, USA) equilibrated and eluted with 200 mM ammonium acetate buffer (pH 5.0), as described by Mort et al. [20]. Fractions of 1.5 mL were collected at a flow rate of 36 mL h^−1^. The uronic acid content in each fraction was determined using the m-hydroxydiphenyl method [18].

Hemicelluloses soluble in 4% and 24% KOH were applied to a Sepharose CL-6B-100 column equilibrated and eluted with 0.1 M NaOH as described by Maclachlan and Brady [17]. The total sugar content of each 1.5 mL fraction was determined using the phenol–sulfuric acid method [19].

### 2.8. Statistical Analysis

Prior to ANOVA, the assumptions of normality and homogeneity of variance were assessed for all datasets using the Shapiro–Wilk test and Levene’s test, respectively. Statistical analyses were performed using a two-way analysis of variance (ANOVA) with cultivar and treatment as factors. When significant effects were detected, post hoc comparisons were performed using the Tukey–Kramer HSD test. Student’s *t*-test was applied for direct pairwise comparisons between two cultivars. Statistical significance was set at *p* < 0.05, with *p* < 0.01 considered highly significant. All analyses were conducted using JMP software (version 5.0.1; SAS Institute Inc., Cary, NC, USA).

## 3. Results

### 3.1. Fruit Quality

Fruit quality parameters are summarized in Table 1. No significant differences were observed among the cultivars in terms of fresh weight, fruit length, fruit diameter, or L/D ratio. Skin color parameters (L*, a*, and b*) differed significantly among cultivars (Figure 1); ‘Fuji’ showed the highest a* and lowest L* values, consistent with its red skin coloration, whereas ‘Toki’ exhibited the highest L* value and ‘Bramley’s Seedling’ showed the lowest a* and highest b* values, reflecting its green–yellow skin. ‘Bramley’s Seedling’ alone showed a high starch index, whereas the other two cultivars showed low starch index values. ‘Bramley’s Seedling’ also exhibited higher ethylene production than the other two cultivars. These differences in starch index and ethylene production indicate that physiological maturity was not fully equivalent among the three cultivars at the time of harvest. The SSC of ‘Bramley’s Seedling’ was lower than that of the other two cultivars. Malic acid content varied significantly among the cultivars; it was highest in ‘Bramley’s Seedling’, lowest in ‘Toki’, and intermediate in ‘Fuji’.

### 3.2. Flesh Firmness and Texture Analysis

**Figure 2:**
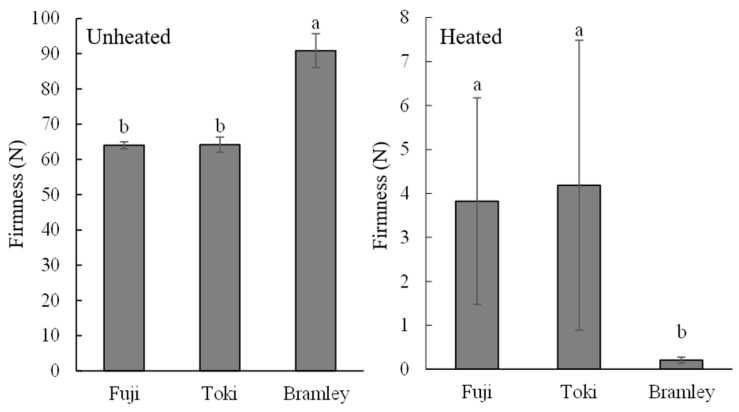
Flesh firmness (N) of ‘Fuji’, ‘Toki’, and ‘Bramley’s Seedling’ (Bramley) in unheated and heated samples. Different letters indicate a significant difference by the Tukey–Kramer HSD test at the 5% level (*n* = 6).

The firmness of unheated flesh was higher in ‘Bramley’s Seedling’ (approximately 90 N) than in ‘Fuji’ and ‘Toki’ (approximately 64 N) (Figure 2). In contrast, for heated flesh, ‘Bramley’s Seedling’ exhibited markedly lower firmness after heating compared with the other two cultivars.

Texture profile analysis further supported these findings (Table 2). Comparison of ‘Toki’ and ‘Bramley’s Seedling’ revealed that ‘Bramley’s Seedling’ exhibited higher viscosity and lower cohesiveness, gumminess, and chewiness values. In particular, gumminess and chewiness showed the greatest differences between the two cultivars (Table 2). We attempted to measure ‘Fuji’ under the same conditions, but the force exceeded the instrument’s measurement limit (50 N), so we were unable to obtain any results. In other words, it is clear that it maintained a structural integrity greater than that of ‘Toki’.

### 3.3. Structural Changes Following Heating Treatment

**Figure 3:**
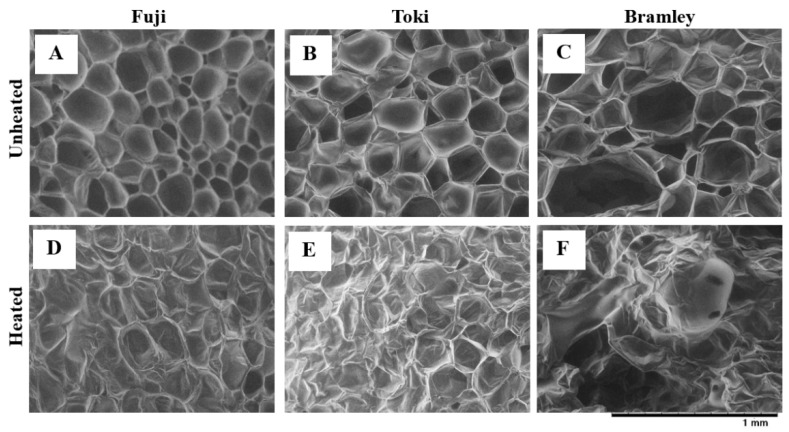
Cell structure of apple flesh before and after heating in three apple cultivars. (**A**,**D**) ‘Fuji’; (**B**,**E**), ‘Toki’; (**C**,**F**), ‘Bramley’s Seedling’; (Bramley). (**A**–**C**) unheated; (**D**–**F**) heated.

Scanning electron microscopy revealed distinct differences in cell structure between unheated and heated samples among the three cultivars (Figure 3). In unheated samples, parenchyma cells of all three cultivars appeared intact with well-defined cell boundaries (Figure 3A–C). Cells of ‘Fuji’ and ‘Toki’ were uniformly small, whereas those of ‘Bramley’s Seedling’ were more heterogeneous in size and shape. After heating, structural disintegration of parenchyma cells was observed in all three cultivars, although the extent varied considerably (Figure 3D–F). ‘Bramley’s Seedling’ exhibited extensive cell disintegration, with most cells losing their defined boundaries and large intercellular spaces developing throughout the tissue. In contrast, ‘Fuji’ and ‘Toki’ largely maintained their cellular integrity after heating, with ‘Fuji’ in particular retaining notably uniform cell morphology.

### 3.4. Cell Wall-Bound Ca Content

Among the three cultivars examined, there was no difference in the Ca content bound to flesh cell walls (Table 3).

### 3.5. Cell Wall Components

The EIS content of both unheated and heated samples in ‘Bramley’s Seedling’ was higher than that in ‘Fuji’ and ‘Toki’ (Table 4). Among the three cultivars examined, only ‘Bramley’s Seedling’ showed an increase in EIS content after heating. In both unheated and heated samples, the starch content of ‘Bramley’s Seedling’ was higher than that of the other two cultivars.

The water-soluble pectin content in unheated flesh was similar among the three cultivars, and heating treatment induced an increase in this fraction. However, the magnitude of this increase differed markedly among cultivars: 421.7% in ‘Bramley’s Seedling’ compared with approximately 239% in the other two cultivars. The EDTA-soluble pectin in unheated flesh was the highest in ‘Fuji’, followed by ‘Bramley’s Seedling’, and ‘Toki.’ The content decreased after heating in all three cultivars, although the degree differed among cultivars: the decrease rates for ‘Bramley’s Seedling’, ‘Fuji’, and ‘Toki’ were 81.3%, 38.9%, and 27.3%, respectively. The Na_2_CO_3_-soluble pectin in unheated flesh showed a similar cultivar-dependent pattern to that of EDTA-soluble pectin, with ‘Fuji’ having the highest content, followed by ‘Bramley’s Seedling’ and ‘Toki’. Heating treatment reduced the content of this fraction; however, the degree of reduction was greater for ‘Bramley’s Seedling’ (68.3%) than for the other two cultivars (approximately 25%). The decrease in ‘Toki’ following heating was not significant, possibly reflecting its lower initial Na_2_CO_3_-soluble pectin content.

The 4% KOH-soluble hemicellulose in unheated flesh was the highest in ‘Fuji’, followed by ‘Toki’ and ‘Bramley’s Seedling’. Following heating, the 4% KOH-soluble hemicellulose content decreased in ‘Fuji’ and ‘Toki’, but not in ‘Bramley’s Seedling’, which had a lower initial content. The 24% KOH-soluble hemicellulose content in unheated flesh was higher in both ‘Bramley’s Seedling’ and ‘Toki’ than in ‘Fuji.’ Among the three cultivars, only ‘Bramley’s Seedling’ showed a significant decrease in 24% KOH-soluble hemicellulose content after heating.

The cellulose content of unheated flesh was comparable among the three cultivars, and heating treatment reduced this content in all three cultivars. However, ‘Bramley’s Seedling’ showed a substantially greater reduction in cellulose content (50.5%) than ‘Fuji’ and ‘Toki’ (approximately 25%), with the decrease in ‘Toki’ being non-significant.

### 3.6. Molar Mass Distribution

**Figure 4:**
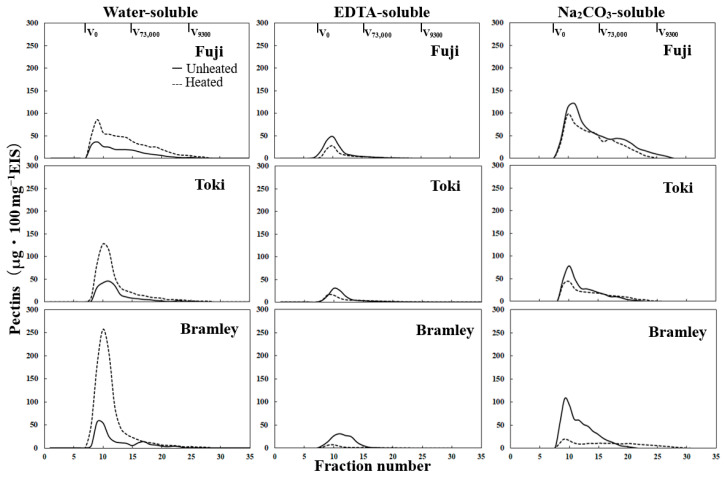
Molar mass distribution profiles of water-, EDTA-, and Na_2_CO_3_-soluble pectic fractions from ‘Fuji’, ‘Toki’, and ‘Bramley’s Seedling’ (Bramley) in unheated and heated samples. The vertical axis represents pectin content (μg 100 mg^−1^ EIS).

The molar mass distribution profiles of water-, EDTA-, and Na_2_CO_3_-soluble pectins are shown in Figure 4. Profiles normalized to the total uronic acid content for cultivar-independent comparison are provided in Figure S1. In the water-soluble pectin fraction of the unheated flesh, ‘Fuji’ and ‘Toki’ showed a broad molar mass distribution extending from high to low-molar-mass regions. In contrast, ‘Bramley’s Seedling’ exhibited a peak in the high-molar-mass region, along with a smaller peak in the low-molar-mass region. After heating, all three cultivars exhibited distinct peaks in the high-molar-mass region, with the peak intensity of ‘Bramley’s Seedling’ being greater than that of the other two cultivars. The peak in the high-molar-mass region of ‘Fuji’ was smaller than that of the other two cultivars, and the polymers were widely distributed in the low-molar-mass region.

In the EDTA-soluble pectin fraction of unheated flesh, all three cultivars showed a sharp peak in the high-molar-mass region; however, ‘Bramley’s Seedling’ had a slightly broader distribution than the other two cultivars. Heating reduced the high-molar-mass peak in all three cultivars, although the reduction was considerably greater in ‘Bramley’s Seedling’ than in the other two cultivars.

In the Na_2_CO_3_-soluble pectin fraction of unheated flesh, all three cultivars exhibited similar molar mass distributions, characterized by a dominant peak in the high-molar-mass region, followed by a gradual decrease toward the low-molar-mass region. However, ‘Toki’ exhibited a somewhat smaller peak, and ‘Bramley’s Seedling’ showed a shift toward the high-molar-mass region. After heating, the changes in ‘Bramley’s Seedling’ were markedly more pronounced than those in the other two cultivars, with a substantial reduction in the high-molar-mass peak and a corresponding increase in the low-molar-mass region. Similar but less pronounced changes were observed in ‘Toki’. In ‘Fuji’, no substantial changes in molar mass distribution were observed after heating, with only a slight decrease across all molar mass regions. Across all three pectin fractions, heating-induced changes in molar mass distribution were consistently most pronounced in ‘Bramley’s Seedling’.

**Figure 5:**
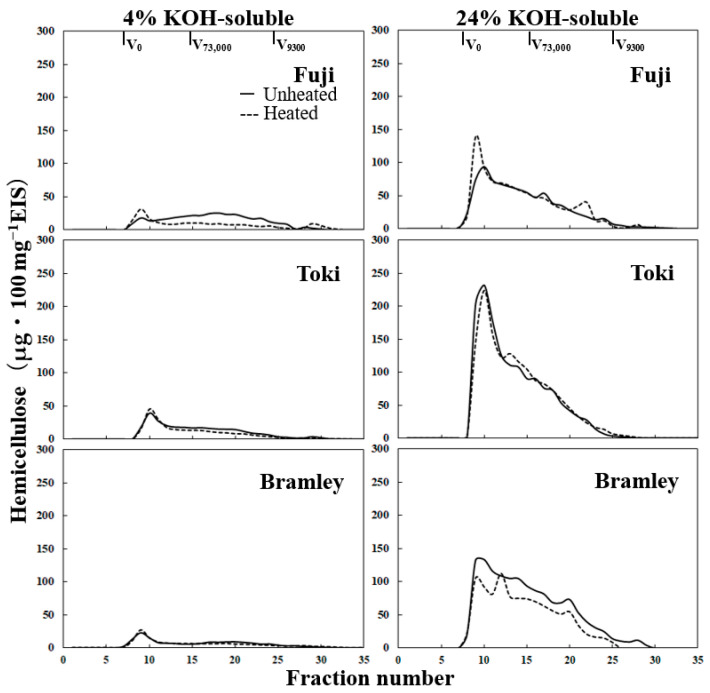
Molar mass distribution profiles of 4% KOH-, and 24% KOH-soluble hemicelluloses from ‘Fuji’, ‘Toki’, and ‘Bramley’s Seedling’ (Bramley) in unheated and heated samples. The vertical axis represents hemicellulose content (μg 100 mg^−1^ EIS).

In the 4% KOH-soluble hemicellulose fraction of unheated flesh, ‘Toki’ and ‘Bramley’s Seedling’ exhibited peaks in the high-molar-mass region, followed by a gradual decrease toward the low-molar-mass region (Figure 5 and Figure S2). The 4% KOH-soluble hemicellulose fraction of unheated ‘Fuji’ flesh showed a distinct molar mass distribution, with a broad peak in the mid-molar-mass region rather than in the high-molar-mass region. After heating, this broad mid-molar-mass peak disappeared, and a distinct peak in the high-molar-mass region emerged. In the 24% KOH-soluble hemicellulose fraction, all cultivars exhibited a dominant peak in the high-molar-mass region, followed by a gradual decrease toward lower molar masses, with no substantial changes observed after heating.

## 4. Discussion

This study compared cultivar-dependent patterns of pectin depolymerization and cell wall disassembly between a traditional cooking apple cultivar (‘Bramley’s Seedling’) and dessert cultivars (‘Fuji’ and ‘Toki’) during thermal processing. ‘Bramley’s Seedling’ is widely recognized for its pronounced flesh breakdown during cooking [21], yet the physiological and structural mechanisms underlying cultivar-dependent differences in heat-induced tissue disintegration remain incompletely understood [4,11]. ‘Bramley’s Seedling’ exhibited severe tissue disintegration after brief heating, whereas ‘Fuji’ and ‘Toki’ largely maintained their structural integrity. Notably, the initial firmness of raw fruit did not predict the extent of softening after heating, indicating that thermal processing behavior cannot be inferred solely from fresh texture.

### 4.1. Heat-Induced Softening and Cultivar-Specific Cellular Responses

Heating resulted in a substantial decrease in flesh firmness in all cultivars; however, the magnitude of softening varied considerably. ‘Bramley’s Seedling’, which exhibited the highest firmness in the raw state, showed the lowest firmness after heating, whereas ‘Fuji’ and ‘Toki’ retained relatively higher firmness (Figure 2). This inverse relationship between raw and post-heating firmness is consistent with previous findings for thermally processed apples [14,22,23]. For example, Bourles et al. [22] reported that firmer apple cultivars at harvest exhibited greater softening following vacuum cooking than those with lower initial firmness. Microscopic analysis revealed that cellular morphology was comparable among cultivars before heating (Figure 3A–C). After heating, however, extensive cell collapse and tissue disintegration occurred exclusively in ‘Bramley’s Seedling’ (Figure 3D–F). In contrast, the cellular structure of ‘Fuji’ and ‘Toki’ remained largely intact. These findings indicate that ‘Bramley’s Seedling’ is intrinsically susceptible to heat-induced cellular disintegration.

### 4.2. Initial Cell Wall Composition Is Insufficient to Explain Cultivar-Dependent Thermal Breakdown

Fruit firmness is determined by the composition and structural organization of the cell wall, in which cellulose, hemicellulose, and pectin each play distinct roles [5,11,24,25]. In peach, tissue firmness has been positively correlated with higher proportions of structurally bound polysaccharides and lower water-soluble pectin content [7]. All cultivars showed similarly high cellulose and low water-soluble pectin contents before heating, characteristics generally associated with firm tissues [7] (Table 4). Some cultivar-dependent differences were observed in hemicellulose and EDTA- and Na_2_CO_3_-soluble pectin fractions. However, these differences did not correspond directly with raw flesh firmness. These results indicate that cultivar-dependent thermal breakdown cannot be explained by initial cell wall polysaccharide composition alone, but rather reflects dynamic modifications of these components during heating.

### 4.3. Solubilization and Depolymerization of Pectin During Heating

Pectin solubilization and depolymerization are well-documented responses to both fruit maturation [26,27], ripening [28,29] and thermal processing [12,14]. ‘Bramley’s Seedling’ exhibited a markedly greater increase in water-soluble pectin content after heating than ‘Fuji’ and ‘Toki’ (Table 4), indicating enhanced pectin solubilization. This increase was accompanied by a marked decrease in EDTA- and Na_2_CO_3_-soluble pectin fractions, suggesting that these ionically bound and alkali-labile pectic fractions were converted into water-soluble forms during heating.

Only ‘Bramley’s Seedling’ exhibited a pronounced shift from high- to low-molar-weight pectin within the Na_2_CO_3_-soluble fraction (Figure 4 and Figure S1). When expressed as relative proportions, this shift was specific to the Na_2_CO_3_-soluble pectin of ‘Bramley’s Seedling’. A similar pattern of reduced molar mass in the Na_2_CO_3_-soluble fraction has been associated with softening in melting-type peach cultivars during ripening [7], suggesting that depolymerization of this fraction may be a conserved feature of tissue softening across both developmental and thermal processes. Thermal processing studies have shown that heating can induce substantial depolymerization of pectic polysaccharides, resulting in a shift from high- to low-molar-mass polymers and contributing to softening of plant tissues [30].

The observed depolymerization may reflect both enzymatic and non-enzymatic processes. However, in the present study, since the temperature was rapidly raised to 105 °C under high-pressure conditions, the cell wall-modifying enzymes lost their activity within a short period of time. Therefore, non-enzymatic factors play a major role in fruit softening. Non-enzymatic softening of fruit has been attributed to β-elimination of homogalacturonan as a primary mechanism of pectin degradation [11]. The resulting reduction in molar mass within the Na_2_CO_3_-soluble fraction may therefore contribute to the loss of structural integrity and rapid tissue disintegration observed in ‘Bramley’s Seedling’. Although we were unable to perform the analysis due to experimental limitations, analyzing the degree of methyl esterification and acetylation [4,25] would likely be helpful in elucidating the precise mechanism of fruit softening.

### 4.4. Contributions of Hemicellulose and Cellulose to Tissue Integrity

Hemicellulose plays a key role in cross-linking cellulose microfibrils and maintaining the mechanical integrity of the cell wall matrix [6]. Ng and Waldron [31] reported that decreases in hemicellulose and cellulose are associated with thermal softening in carrot, in addition to pectin modification. Cellulose content decreased in all cultivars following heating, with the greatest reduction observed in ‘Bramley’s Seedling’ (Table 4). Given that cellulose is the primary load-bearing component of the cell wall [6], its preferential reduction in ‘Bramley’s Seedling’ may contribute to the pronounced loss of mechanical strength observed in this cultivar. In contrast, the molar mass distribution of hemicellulose fractions showed minimal changes upon heating, as observed in both absolute quantities (Figure 5) and normalized profiles (Figure S2), suggesting that hemicellulose depolymerization is not a major contributor to thermal tissue breakdown under the present conditions.

There are limited reports demonstrating direct thermal degradation of cellulose under conditions comparable to those used in the present study. Therefore, the possibility cannot be excluded that other factors, such as polysaccharide redistribution, may have contributed to the apparent changes in cellulose content. To verify the hypothesis presented here, additional analyses, such as X-ray diffraction, which can evaluate crystallinity and structural organization [11,32], will likely be necessary.

### 4.5. Role of Calcium-Mediated Pectin Interactions

Calcium-mediated cross-linking has been reported to suppress pectin solubilization and tissue softening during heating [33,34]. However, no significant differences in cell wall-bound Ca content were detected among cultivars (Table 3). These results suggest that cell wall-bound Ca content alone is insufficient to explain cultivar-dependent differences in heat-induced softening.

The larger and more heterogeneous cell structure of ‘Bramley’s Seedling’ observed before heating (Figure 3) may itself contribute to reduced mechanical stability under thermal stress, independently of Ca-mediated cross-linking. Taken together, these observations suggest that structural features of the cell wall—such as pectin architecture and cellular organization—rather than Ca content, are more likely to govern susceptibility to heat-induced tissue breakdown.

### 4.6. Integration of Microstructural and Textural Changes During Heating

The two heating methods used in this study—microwave heating for texture and microstructural analyses, and autoclaving for cell wall fractionation—provided complementary perspectives on tissue-level and biochemical responses to heat. Consistent with observations in other thermally processed plant tissues such as carrot [31,35], texture changes in the present study were closely associated with modifications in cell wall polysaccharides. In ‘Bramley’s Seedling’, a marked loss of firmness after heating was accompanied by extensive tissue disintegration observed microscopically and an increase in viscosity. This structural collapse was associated with pronounced pectin solubilization, depolymerization of the Na_2_CO_3_-soluble pectin fraction, and reductions in hemicellulose and cellulose content. In contrast, ‘Fuji’ and ‘Toki’ retained relatively stable firmness after heating, consistent with limited changes in cell wall composition. These results indicate that the degree of pectin solubilization and depolymerization is closely linked to textural outcomes, and that cultivar-dependent differences in cell wall modification determine the textural quality of thermally processed apple fruit.

### 4.7. Limitations and Future Perspectives

While this study provides novel insights into variety-dependent cell wall modifications during heating, its limitations and future directions can be summarized under three points.

First, with respect to mechanistic limitations, future work should evaluate the degree of methyl esterification and acetylation—both of which influence susceptibility to thermal degradation—as well as the activity and expression levels of cell wall-modifying enzymes. As the present study was unable to fully establish a causal relationship between the observed compositional changes and the underlying biological mechanisms, these aspects remain important topics for future investigation.

Second, regarding sampling, although fruits were harvested at the commercially recommended maturity stage, the starch index and content data obtained in this study revealed that physiological maturity varies among individual cultivars, particularly in ‘Bramley’s Seedling’. While the effect of maturity was not directly evaluated here, preliminary observations suggest that ‘Bramley’s Seedling’ exhibits similar heat-induced softening behavior across different maturity stages (unpublished data). Future studies should verify these findings using samples at a uniform stage of physiological maturity.

Third, regarding the generalizability of the findings, since only three cultivars were examined, future research should encompass a broader range of apple cultivars to characterize the extent of thermal degradation more comprehensively, thereby providing a basis for evaluating thermal softening susceptibility and informing breeding programs for novel cultivars.

Despite these limitations, the present data clearly demonstrate that differential pectin solubilization and depolymerization are key determinants of thermal flesh breakdown in apple fruit.

## 5. Conclusions

This study demonstrated that cultivar-dependent differences in the thermal softening of apple fruit are closely associated with distinct patterns of cell wall polysaccharide modification. ‘Bramley’s Seedling’ exhibited markedly greater pectin solubilization and depolymerization during heating compared with ‘Fuji’ and ‘Toki’, accompanied by marked reductions in cellulose content and pronounced shifts in the molar mass distribution of pectin. These findings suggest that thermal breakdown in apple fruit is not solely governed by pectin modification but also by cultivar-specific patterns of cell wall disassembly. In particular, the associated changes in pectin and cellulose observed in ‘Bramley’s Seedling’ suggest a distinct mode of cell wall disassembly underlying its characteristic rapid tissue disintegration during heating. These findings provide a structural and compositional basis for understanding why ‘Bramley’s Seedling’ undergoes rapid tissue disintegration during heating, and may inform the selection of apple cultivars for specific culinary and industrial applications.

## Figures and Tables

**Table 1:** Fruit quality of ‘Fuji’, ‘Toki’, and ‘Bramley’s Seedling’ (Bramley) at commercial harvest maturity.

Cultivar	Fruit Weight (g)		Length (mm)		Diameter (mm)		L/D Ratio		Skin Color						Starch Content Index		Ethylene Production (µL·kg^−1^·h^−1^)		Soluble Solids Content (Brix°)		Malic Acid Content (mg·100^−1^ mL)	
									L*		a*		b*									
Fuji	363.4	a ^z^	86.1	a	92.0	a	0.94	a	42.8	c	32.3	a	19.4	c	1.7	b	1.6	b	15.4	a	0.4	b
Toki	356.0	a	83.6	a	94.8	a	0.88	a	73.3	a	−6.4	b	38.5	b	1.0	b	2.2	b	14.8	a	0.3	c
Bramley	359.3	a	85.5	a	93.0	a	0.92	a	62.3	b	−16.8	c	47.6	a	3.5	a	7.6	a	10.8	b	1.3	a

^z^ Different letters indicate a significant difference by the Tukey–Kramer HSD test at the 5% level (*n* = 6).

**Table 2:** Texture profile analysis of heated flesh of ‘Toki’ and ‘Bramley’s Seedling’ (Bramley).

Cultivar	Viscosity (N·m^2^·10^−3^)		Cohesiveness		Adhesiveness (J·m^3^·10^−3^)		Resilience		Gumminess (N·m^2^·10^−3^)		Chewiness (N·m^2^·10^−3^)	
Toki	1.1	*^z^	0.5	*	1.0	ns	0.8	ns	22.3	**	17.0	**
Bramley	1.7		0.2		1.2		0.7		4.5		3.2	

^z^, **, *, and ns indicate significant differences at the 1% and 5% levels, and no significant difference, respectively, by Student’s *t*-test (*n* = 6).

**Table 3:** Cell wall-bound calcium contents of ‘Fuji’, ‘Toki’, and ‘Bramley’s Seedling’ (Bramley).

Cultivar	Calcium Content (μg·g^−1^ DW)
Fuji	66.6 a ^z^
Toki	62.8 a
Bramley	70.1 a

^z^ Different letters indicate a significant difference by Tukey–Kramer’s HSD test at the 5% level (*n* = 3).

**Table 4:** Ethanol insoluble solid (EIS), starch content, and cell wall components contents of unheated and heated samples of three apple cultivars.

Cultivar	Treatment	EIS Content (mg·10 g^−1^ FW)		Starch Content (mg·100 mg^−1^ EIS)		Pectin Content (mg·100 mg^−1^ EIS)						Hemicellulose Content (mg·100 mg^−1^ EIS)				Cellulose Content (mg·100 mg^−1^ EIS)	
						Water		EDTA		Na_2_CO_3_		4% KOH		24% KOH			
Fuji	Unheated	153.1	c ^z^	1.4	c	2.6	c	1.8	a	7.5	a	3.4	a	8.0	b	51.0	a
	Heated	164.7	c	1.3	c	6.2	b	1.1	c	5.8	b	2.0	d	8.8	bc	37.3	c
Toki	Unheated	167.4	c	1.9	b	2.3	c	1.1	c	3.7	c	2.8	b	14.6	a	50.7	ab
	Heated	172.3	c	1.4	c	5.5	b	0.8	d	2.7	cd	2.4	c	14.4	a	38.7	bc
Bramley	Unheated	214.9	b	2.6	a	2.3	c	1.6	b	6.3	b	1.7	de	14.0	a	43.0	abc
	Heated	266.0	a	2.6	a	9.7	a	0.3	e	2.0	d	1.5	e	10.3	b	21.3	d
ANOVA																	
Cultivar (A)		**^y^		**		**		**		**		**		**		**	
Treatment (B)		**		*		**		**		**		**		**		**	
A × B		*		*		**		**		**		**		**		ns	

^z^ Different letters indicate a significant difference by Tukey–Kramer’s HSD test at the 5% level (*n* = 3). ^y^, **, *, and ns indicate significant differences at the 1% and 5% levels, and no significant difference, respectively, by two-way ANOVA.

## Data Availability

The data presented in this study are available upon request from the corresponding author. The data are not publicly available due to institutional policies.

## Supplementary Materials

The following supporting information can be downloaded at: https://www.mdpi.com/article/10.3390/foods15081375/s1, Figure S1: Molar mass distribution profiles of water-, EDTA-, and Na_2_CO_3_-soluble pectic fractions from ‘Fuji’, ‘Toki’, and ‘Bramley’s Seedling’ (Bramley) in unheated and heated samples. Figure S2: Molar mass distribution profiles of 4% KOH-, and 24% KOH-soluble hemicelluloses from ‘Fuji’, ‘Toki’, and ‘Bramley’s Seedling’ (Bramley) in unheated and heated samples.

## Abbreviations

The following abbreviations are used in this manuscript:

DAFB	Days after full bloom
SSC	Soluble solids content
