# Supplementary material for: Cultivar-Dependent Thermal Flesh Breakdown in Apple Associated with Cell Wall Polysaccharide Modification, with Pronounced Effects in Cooking Apple ‘Bramley’s Seedling’
Source: Foods. 2026 Apr 15;15(8):1375. doi: 10.3390/foods15081375 (PMC13115144; doi:10.3390/foods15081375)
Supplement: Supplementary file 1 [file foods-15-01375-s001.zip › foods-4238992-supplementary.pdf]

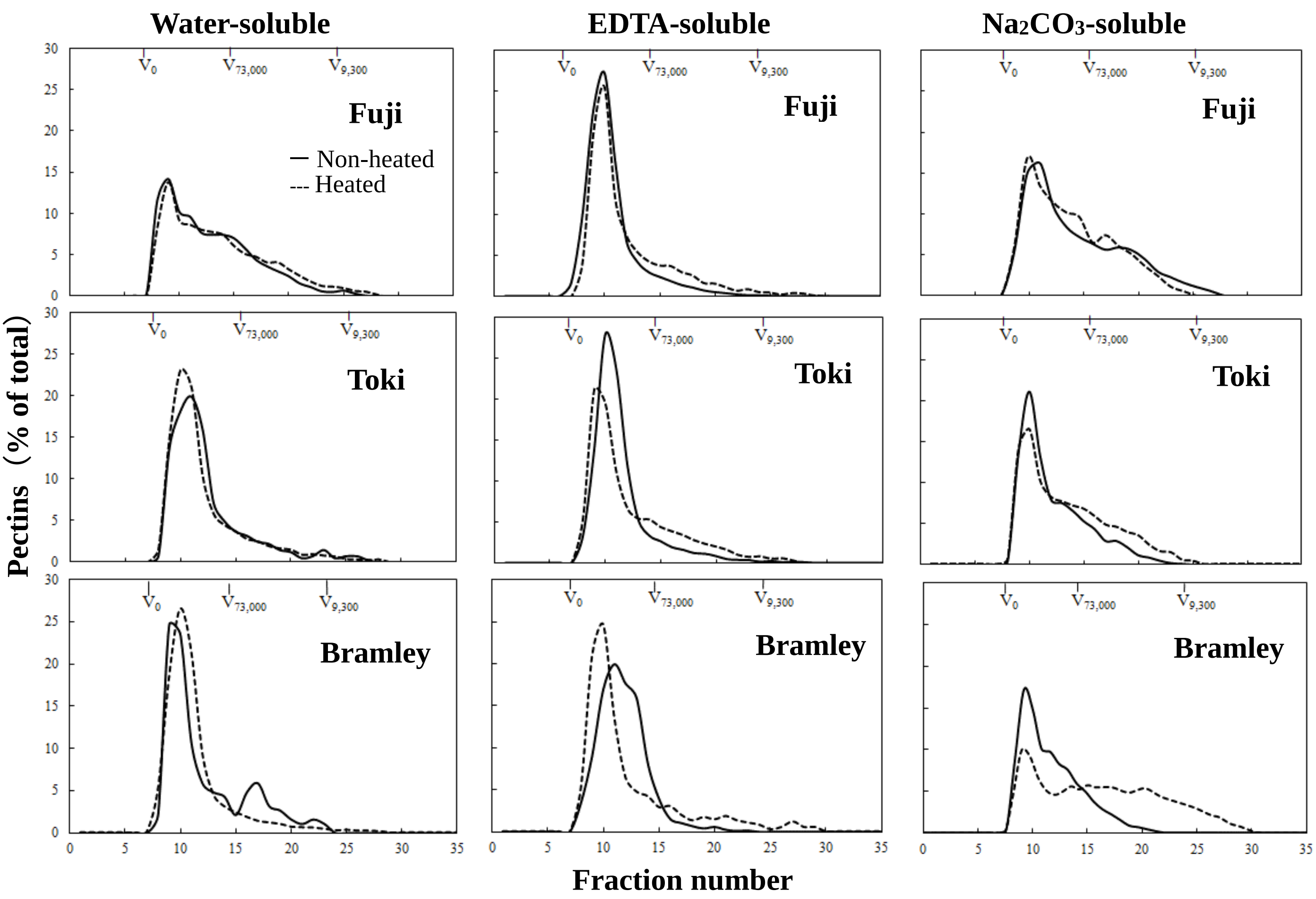

Figure S1. Molar mass distribution profiles of water-, EDTA-, and Na_2CO_3-soluble pectic fractions from 'Fuji', 'Toki', and 'Bramley's Seedling' (Bramley) in unheated and heated samples. The vertical axis represents the relative pectin content (% of total) to allow cultivar-independent comparison of distribution patterns.

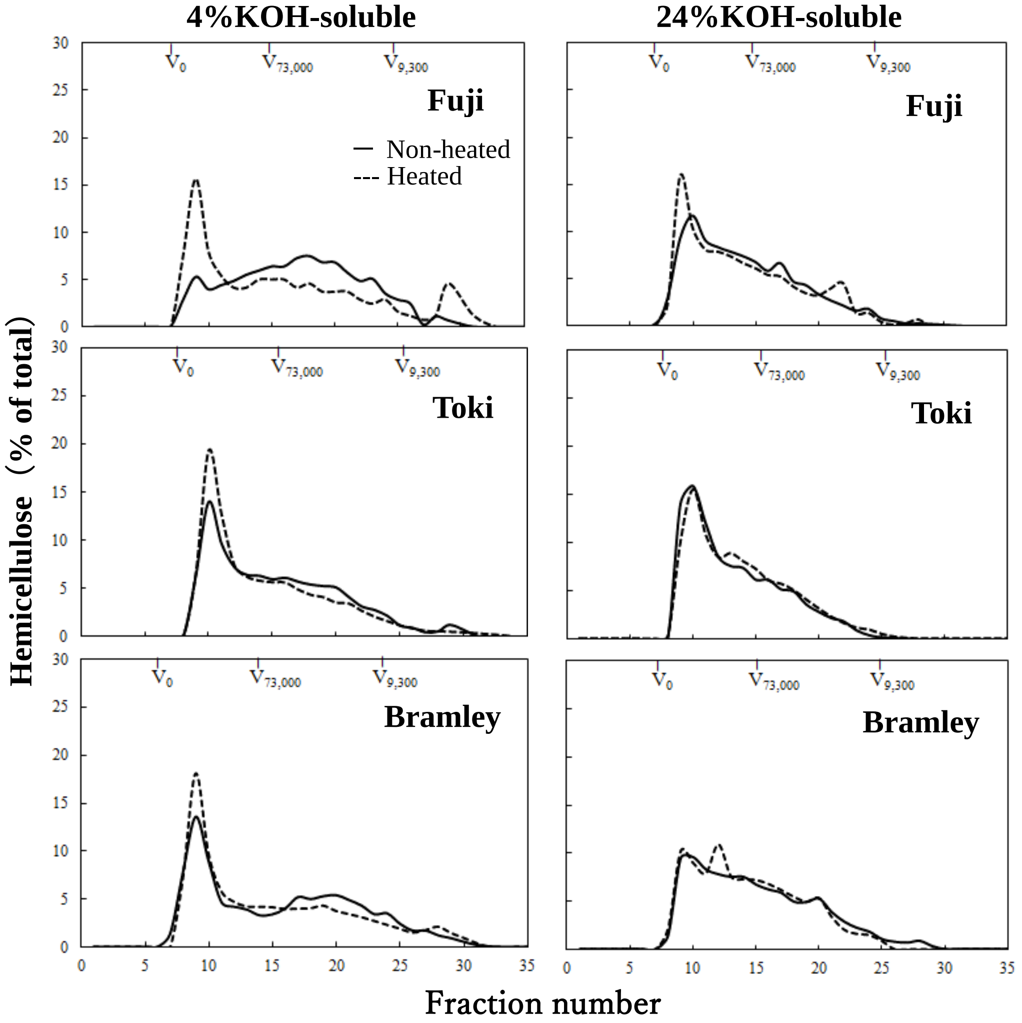

Figure S2. Molar mass distribution profiles of 4% KOH-, and 24% KOH-soluble hemicelluloses from 'Fuji', 'Toki', and 'Bramley's Seedling' (Bramley) in unheated and heated samples. The vertical axis represents the relative hemicellulose content (% of total) to allow cultivar-independent comparison of distribution patterns.
